# Population Genetics and Reproductive Strategies of African Trypanosomes: Revisiting Available Published Data

**DOI:** 10.1371/journal.pntd.0003985

**Published:** 2015-10-22

**Authors:** Mathurin Koffi, Thierry De Meeûs, Modou Séré, Bruno Bucheton, Gustave Simo, Flobert Njiokou, Bashir Salim, Jacques Kaboré, Annette MacLeod, Mamadou Camara, Philippe Solano, Adrien Marie Gaston Belem, Vincent Jamonneau

**Affiliations:** 1 Université Jean Lorougnon GUEDE, UFR Environnement-Santé, Laboratoire des Interactions Hôte-Microorganismes-Environnement et Evolution (LIHME), Daloa, Côte d'Ivoire; 2 IRD, UMR 177 IRD-CIRAD INTERTRYP, Centre International de Recherche-Développement sur l’Elevage en zone Subhumide (CIRDES), Bobo-Dioulasso, Burkina-Faso; 3 IRD, UMR177 IRD-CIRAD INTERTRYP, Campus International de Baillarguet, TA A-17/G, Montpellier, France; 4 Programme National de Lutte contre la Trypanosomiase Humaine Africaine, Conakry, Guinée; 5 University of Dschang, Faculty of Sciences, Department of Biochemistry, Dschang, Cameroon; 6 University of Yaoundé 1, Faculty of Sciences, Department of Animal Biology and Physiology, Yaoundé, Cameroon; 7 University of Khartoum, Department of Parasitology, Faculty of Veterinary Medicine, Khartoum North, Sudan; 8 Parasites, Vectors and Vector-borne Diseases, Agricultural Research Council-Onderstepoort Veterinary Institute, Onderstepoort, South Africa; 9 Université Polytechnique de Bobo-Dioulasso, UFR Sciences et Techniques, Bobo-Dioulasso, Burkina Faso; 10 University of Glasgow, Wellcome Centre for Molecular Parasitology, Henry Wellcome Building of Comparative Medicine, Glasgow, United Kingdom; Yale University, UNITED STATES

## Abstract

Trypanosomatidae are a dangerous family of Euglenobionta parasites that threaten the health and economy of millions of people around the world. More precisely describing the population biology and reproductive mode of such pests is not only a matter of pure science, but can also be useful for understanding parasite adaptation, as well as how parasitism, specialization (parasite specificity), and complex life cycles evolve over time. Studying this parasite’s reproductive strategies and population structure can also contribute key information to the understanding of the epidemiology of associated diseases; it can also provide clues for elaborating control programs and predicting the probability of success for control campaigns (such as vaccines and drug therapies), along with emergence or re-emergence risks. Population genetics tools, if appropriately used, can provide precise and useful information in these investigations. In this paper, we revisit recent data collected during population genetics surveys of different *Trypanosoma* species in sub-Saharan Africa. Reproductive modes and population structure depend not only on the taxon but also on the geographical location and data quality (absence or presence of DNA amplification failures). We conclude on issues regarding future directions of research, in particular vis-à-vis genotyping and sampling strategies, which are still relevant yet, too often, neglected issues.

## Introduction

African trypanosomes are parasites with a complex life cycle. Apart from *Trypanosoma equiperdum*, which is transmitted sexually [1], they necessarily involve two host species: a vertebrate (mammals, e.g., humans, for the taxa involved in the present paper) and a vector, primarily a tsetse fly, in which sexual recombination events may occur in the salivary glands [2–4]. Some species (*Trypanosoma congolense* and *Trypanosoma vivax*) can alternatively be mechanically transmitted by biting insects (e.g., *Tabanidae*, *Stomoxys*), while *Trypanosoma evansi* has lost its ability for tsetse transmission and is only transmitted mechanically [5–7].

Because of their small size and the difficulty (or impossibility) of using mark-release recapture techniques, population biology of most parasites can only be studied through the analysis of the spatiotemporal distribution of polymorphic genetic markers [8]; trypanosomes are not an exception. These studies are made more difficult by the existence of two relevant compartments, vertebrate and vector. The former offers more opportunities to access population genetics data, whereas, to our knowledge, few or no robust population genetics studies of trypanosomes isolated from tsetse flies have provided interpretable results in terms of demography (but see [9] and our comments on this survey below).

Regarding strains circulating in vertebrates, sound studies of population genetics, mostly based on microsatellite markers, have recently begun to emerge. We hereby propose to revisit all the available published data on trypanosomes isolated in vertebrates and discuss them in light of the known or assumed reproductive system, taking into account the spatiotemporal structure. As much as possible, the technical problems related to the frequently encountered DNA amplification errors will be taken into account. Such problems arise most particularly when parasites are directly amplified from biological fluids without the costly and inconvenient isolation step using, for example, the kit for in vitro isolation of trypanosomes (KIVI) [10] or rodent inoculation (RI) [11]. Note that in [11], based on allozyme profiles, these authors concluded that isolation introduced a strong selection bias because particular profiles appeared to be associated with specific isolation methods. It appeared that this was more the result of a difference in gene expression of trypanosomes inoculated in a mammal (RI) or in an axenic medium (KIVI). Indeed, strains isolated with RI, KIVI, or directly amplified from blood were demonstrated to belong to the same population (no selection bias) [12] and only one strain (probably the major one in multiple infections) was isolated.

Clonal propagation is widespread in parasites and particularly microbes [13,14], making the study of the consequences of clonality on population genetics parameters highly relevant. After an overview of basic concepts in the genetics of clonal populations, this paper revisits the available data on the following: (i) human African trypanosomes responsible for human African trypanosomiasis (HAT, or sleeping sickness) in West and Central Africa (*Trypanosoma brucei gambiense* type 1) and East Africa (*Trypanosoma brucei rhodesiense*) with data from cattle and tsetse flies, and (ii) trypanosomes isolated from domestic ungulates from The Gambia responsible for nagana (*T*. *vivax* and *T*. *congolense* “savannah” type) and trypanosomes isolated from camels in Sudan, outside the tsetse areas, responsible for surra (*T*. *evansi*).

## Overview of Basic Concepts in the Genetics of the Clonal Population

The most important concept to remember is that clones accumulate random mutations, especially on noncoding portions of their genome [8,15,16]. This results in an accumulation of heterozygosity at all loci, because once a homozygous site has experienced mutation, it becomes heterozygous and has very little chance of becoming homozygous again (reverse mutation is unlikely). A homoplasy event (identity between two alleles by random convergent mutation) is therefore the only process that can limit such accumulation; depending on the number of available *K* alleles, more or fewer homozygous genotypes (by state and not by descent) are expected (i.e., 1/*K*) [17]. For example, for SNPs, which generally exhibit two alleles (*K* = 2) [18–20], we expect 50% apparent homozygosity in clones; whereas for reasonably polymorphic microsatellite loci, this proportion will fall (e.g., to 3.3% with *K* = 30) [17]. Relative inbreeding of individuals as compared to subsamples is measured using Wright's [21] *F*_IS_. This *F*_IS_ is equal to zero when reproduction locally follows the panmictic model (fully sexual with a random union of gametes to form zygotes) and becomes negative in highly clonal organisms.

In totally clonal populations, *F*_IS_ directly reflects the size of the population and the mutation rate of genetic markers used [12,16,22,23]. Strongly negative in small populations (-1 being the lowest limit), it will reach less extreme values in larger populations, especially with markers with a high mutation rate and a large number of possible alleles [16]. It has been shown that a low rate of sexual recombination (i.e., the proportion of individuals that are sexually produced at each generation) suffices to significantly change these findings. For a low sex rate (e.g., around 0.1), the proportion of heterozygosity is expected to approach that of panmictic populations. Very low sex rates (0.15 to 0.001) generate negative but also highly variable *F*_IS_ from one locus to another, with some rare loci showing high homozygosity (*F*_IS_>>0) [15,16]. Variance of *F*_IS_ across loci is also expected to occur in case of locus-specific technical problems (e.g., null alleles). This explains why it is important to detect the presence of technical problems during a PCR at one or a few loci. Because it leads affected loci to display a false homozygous profile. It will thus increase the *F*_IS_ estimated at that locus over the value observed at unaffected loci and lead to erroneous conclusions on the reproductive mode [19].

If mutation within the region flanking the targeted locus affects the sector where the PCR primer should hybridize, it can generate null alleles (alleles that are not amplified by the PCR reaction). This generates fictitious homozygous genotypes (genotypes heterozygous for the null allele appear homozygous for the amplified allele) [8].

Limited DNA quantities can also lead to amplification failures of one or both alleles as a result of competition for the Taq polymerase. This is known as allelic dropout, which can also generate substantial numbers of false homozygous phenotypes [24].

In any case, the loci involved will present an increase in their *F*_IS_, which may lead to a profile similar to an almost entirely clonal population with very rare events of sexual recombination. Null homozygotes will be seen as missing genotypes (blanks). Allelic dropout should generally not generate missing data, but it can when primers are not perfect and DNA concentrations are very small. Nevertheless, in clonal diploids, the consequences of null alleles and/or allelic dropout cannot be distinguished (e.g., [19]). This is why we prefer the term "amplification problems," because most of the time the true mechanism cannot be precisely identified, the limit between the two is sometimes fuzzy, and the consequences on *F*_IS_ are undistinguishable.

Homoplasy has an insignificant impact on *F*_IS_ (e.g. [25], pages 62–63), even in clonal organisms [17], and can never produce a pattern similar to rare sex or amplification problems.

Sampling design remains a critical issue [8]. This appears even more critical for small organisms that can be subdivided at extremely small scales, such as the individual host [26], and which often display short generation times, like all microbes. Gathering entities from too-distant sampling sites and/or dates will mix individuals that belong to genetically differentiated subpopulations or cohorts into a single and genetically heterogeneous subsample. This phenomenon is called the Wahlund effect, which can significantly alter population genetics estimates and, when combined with clonal propagation, can drive some estimators in unpredictable directions [27]. This is especially true for *F*_IS_, which asymptotically tends toward its panmictic expectation (*F*_IS_ = 0) when there is a strong Wahlund effect in clones [22]. This is also the case for linkage disequilibrium, which can increase with a moderate Wahlund effect or decrease when there is a strong Wahlund effect in clonal populations [27]. For *T*. *b*. *gambiense*, for instance, much less than one year suffices for significant differentiation to appear between two subsamples from the same focus [28]. Hence, pooling strains that were sampled several years apart will necessarily generate strong Wahlund effects with unpredictable, though important, consequences on the behavior of population genetics parameters.

Another signature of clonality is the presence of identical genotypes at several loci, meaning that several individuals display exactly the same genotype at all loci, which are usually called multilocus genotypes (MLGs). If polymorphic enough, six loci should be enough to ascertain this [29,30]. Using this criterion, however, is not without problems. The presence of repeated genotypes may also be generated in strongly subdivided populations with closed reproductive systems such as selfing, or can be hindered by Wahlund effects [31,32]. Random amplification problems (dropout) and multiple infections can also considerably alter the pattern of MLGs.

When a population is clonal, it was shown that estimating *F*_IS_ from a sufficient number of loci (say, six to seven) and out of sufficient sample sizes (say, 10 to 20) can lead to an accurate estimate of immigration and/or clonal sizes [12,23,26]. Of course it is undoubtedly better to gather results from more loci (the more the better). Nevertheless, it is known that *F*_IS_ estimates display little variance across loci in pure clones. This is why this statistic is preferable to *F*_ST_ for estimating clonal sizes; it provides more accurate and less variable inferences [22] and is totally independent of homoplasy (at least close to equilibrium or in reasonably small populations of ≤1,000 individuals) [17]. Clonal size (*N*_*Cl*_) is the number of propagating clones. It is a clonal equivalent to the effective population size defined for sexual populations. Note that effective population sizes can be computed in clonal populations, but provide contradictory results depending on what definition of effective population size is used. Inbreeding effective population size will be increased in clones (infinity in full clones), while variance effective population size will be reduced. When clonal populations are strongly subdivided into numerous subpopulations and the mutation rate *u*<<*m* (migration rate), the number of immigrant clones can accurately be estimated as [22]:

$$N_{Cl}m=-\frac{1+F_{IS}}{4F_{IS}}$$ (Eq. 1)

When *u* cannot be neglected, then this quantity measures the product *N*_*Cl*_ (*m*+*u*).

When working with an isolated population, then clonal size can be estimated as [23]:

$$N_{Cl}=-\frac{1+F_{IS}}{4uF_{IS}}$$ (Eq. 2)

Another useful case is when the system is composed of two subpopulations, because then *N*_*Cl*_ and *m* can be estimated separately as [12]:

$$N_{Cl}=-\frac{1+F_{IS}}{8uF_{IS}}$$ (Eq. 3)

$$m=\frac{1}{2}\left[1-\sqrt{\frac{F_{ST}}{F_{ST}-4uF_{IS}}}\right]$$ (Eq. 4)

where *F*_ST_ is Wright's fixation index [21] of subpopulations as compared to the total population, and measures inbreeding of subsamples as compared to total inbreeding. This last parameter is less accurately estimated than *F*_IS_ in most situations, especially in clonal populations [22].

These methods require almost perfect genotyping (no amplification problems) and, for most of them, a clear idea of the mutation rate of the genetic markers used. Nevertheless, most of these equations will provide useful results, at least for comparison purposes. For instance, with *T*. *b*. *gambiense* in western Africa [12], this has provided strong evidence that substantially more clones were circulating than what could be speculated from human prevalence. This was still true even when using the highest mutation rates usually given for microsatellites (10^−3^). Since lower mutation rates provide a larger estimation of *N*_*Cl*_, this was strong evidence for the existence of numerous invisible trypanosome clones circulating in the foci. Later, it was found that many seropositive patients without visible trypanosomes appeared as trypanotolerant subjects acting as reservoirs for the parasite [28,33,34]. This contributed to at least partly explaining the genetic results. We will discuss this issue further at the end of the paper.

Comparison across studies is difficult if the markers used are different, because different mutation rates will produce different estimates (see Eqs 1–4). This is why it is better to use as many loci of the same type (e.g., microsatellite loci) as possible, so that the mean across loci should provide results that may be compared across loci. Sharing similar loci across studies is, of course, even better. This is often the case for *T*. *brucei* population genetics studies, because few markers are available. This is also true for *T*. *b*. *gambiense* in Guinea, Ivory Coast [12], and the Central African Republic [23], countries that used the same seven loci (see below). When the results from different kinds of markers are compared, the relevance of the comparison will strongly depend on reasonable knowledge (order of magnitude) of the average mutation rate of each type of marker (see below for a comparison between mini- and microsatellite markers and discussion). Nevertheless, when too many amplification problems obscure the results, no inference is possible and, hence, neither is a comparison (see below). It must be added that very small amounts of sexual recombination or unresolved problems of DNA amplifications will eliminate any reasonable inference using either clonal or sexual models.

## Population Genetic Studies of African Trypanosomes

**Fig 1:**
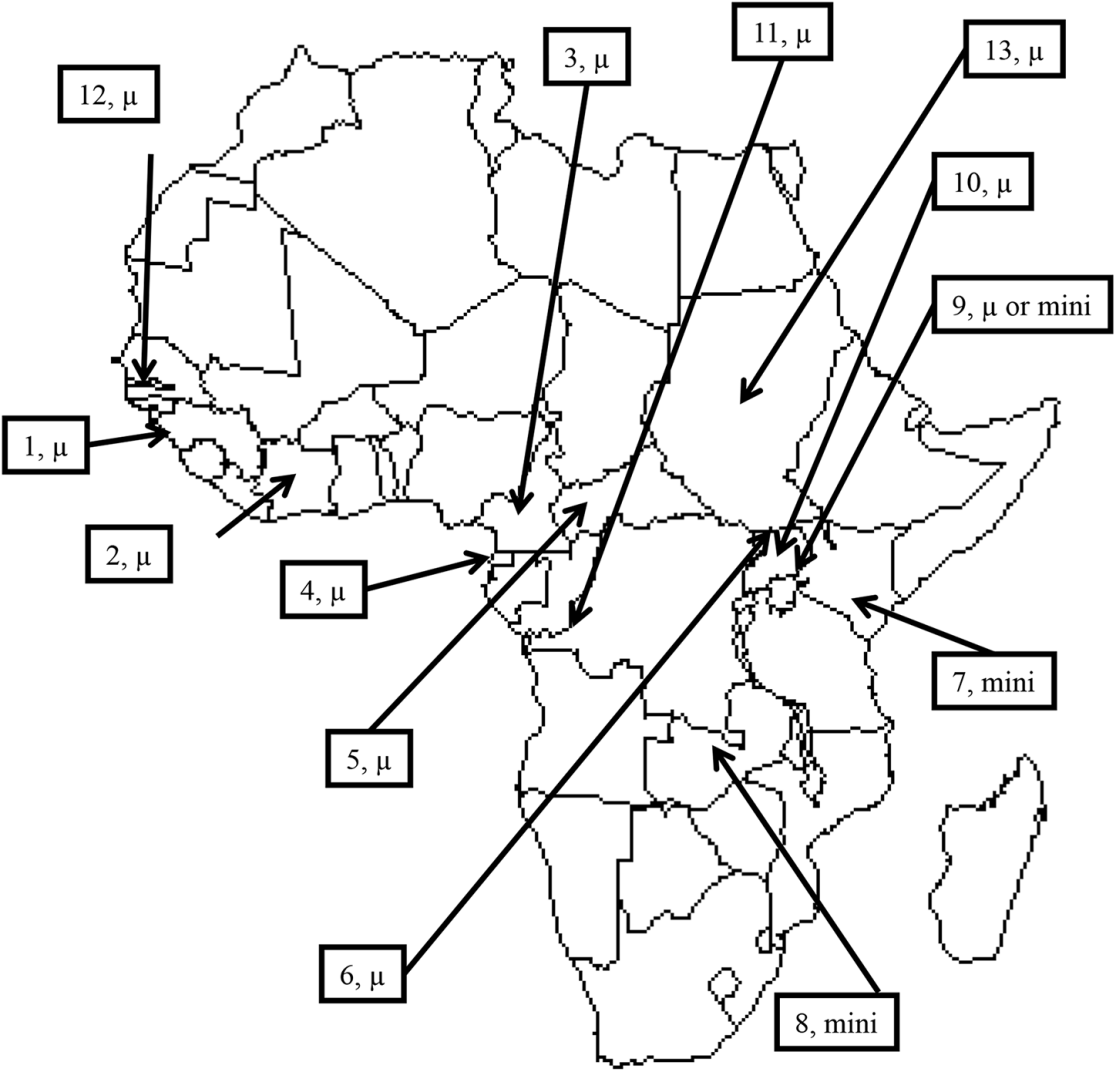
Location of different samples of trypanosomes reanalyzed with population genetics tools for estimating population parameters. 1: Boffa and Dubréka (Guinea) HAT foci. Strains were isolated (I) [12] or directly amplified (D) [28]. 2: Bonon HAT focus (I) (Ivory Coast) [12]. 3: Campo, Bipindi, and Fontem HAT foci (Cameroon), (I) [23], (D) [36], 4: Mbini and Kogo HAT foci (Equatorial Guinea), (I) [23]. 5: Batangafo and Obo HAT foci (Central African Republic), (I) [23]. 6: Omougou HAT foci (Uganda), (I) [23]. 7: Nyanza HAT focus (Kenya), (I) [22,37]. 8: Luangwa HAT focus (Zambia), (I) [22,37]. 9: Busoga HAT focus (Uganda), (I) [22,37], (D) [38]. 10: Soroti HAT focus (Uganda), (D) [38]; 11. Maluku HAT foci (DRC), (D) [36]. 12: Nagana area of Central River District (The Gambia), (D) [5,7]. 13: Surra area of Darfour, Kurdofan, Kassala, Halfa and Showak (Sudan), (D) [39]. μ: Microsatellite genotyping; mini: minisatellite genotyping.

There are few studies on population genetics of African trypanosomes, and even fewer for which the data are available online. Here, we will discuss the published data based on the genotyping of microsatellite and minisatellite markers of different species of trypanosomes from different geographic zones that are presented in Fig 1. We revisited three datasets in which most subsamples were subjected to isolating/amplification techniques (KIVI or RI) before molecular analyses: seven subsamples of *T*. *b*. *gambiense* from humans (Ivory Coast, Guinea, Cameroon, Equatorial Guinea, Central African Republic, Congo, and Uganda) and eight subsamples of *T*. *b*. *rhodesiense* from humans (five from Uganda, Kenya, and Zambia), cattle (two from Uganda) and tsetse flies (one from Uganda) (Fig 1). The trypanosome's DNA from isolated/amplified parasites did not present amplification problems. Nevertheless, strain isolation techniques are very costly in terms of labor and logistics, and all suffer from low success rates, with long adaptation periods needed to propagate the parasite in sufficient numbers [35]. This is why recent surveys use direct DNA PCR amplification from body fluid samples.

We also revisited several sets of data from which trypanosome DNA underwent direct amplification from biological fluids (blood, lymph or cerebrospinal fluid) with no isolation step: one dataset of *T*. *b*. *gambiense* from Guinea, one of *T*. *vivax* from The Gambia, one of *T*. *congolense* ("savannah" type) from The Gambia, and one of *T*. *evansi* from Sudan. One supplementary dataset examined blood sampled from people infected with *T*. *b*. *rhodesiense* from Uganda and Malawi studied at seven microsatellite markers [38]. The results obtained on "forest" type *T*. *congolense* were also introduced for discussion. Another dataset on *T*. *b*. *gambiense* [36], using blood-amplified microsatellite loci, with many obvious amplification problems, was also included.

Datasets were all recoded into appropriate formats with CREATE V 1.1 [40]. Wright's *F*_IS_ and *F*_ST_ analyses were performed with Fstat V 2.9.4 [41] (updated from [42]). The significance of differentiation was tested using Fstat 2.9.4 with the *G*-based test [43] after 10,000 permutations of individuals across subsamples. To compute 95% confidence intervals, we used bootstrap over loci or Jackknife over subsample with Fstat 2.9.4, as described in [8]. When there were fewer than five loci and subsamples, we used bootstrap over alleles with Genetix 4.05 [44]. Regression and testing were undertaken with R 2.14.0 [45]. To compare *F*_IS_ values between two subsamples, we used the exact Wilcoxon signed-rank test for paired data, the pairing unit being each locus. This test was undertaken with R 2.14.0. To test if *F*_IS_ decreases with time in the Bonon focus (Ivory Coast), we used the Page test for ordered alternatives in which matching items are, again, the loci. These two tests are described in Siegel and Castellan's book [46]. Genetic distances were computed with MSA 4.05 [47], and Neighbor Joining Trees built with MEGA 5.2.2 [48]. The robustness of nodes in trees was assessed with 1,000 bootstraps undertaken with PHYLIP V 3.68 [49] with Cavalli-Sforza and Edward's chord distance [50]. In this last case the dataset was converted to PHYLIP format with Convert 1.31 [51].

### Trypanosomes isolated by KIVI or RI

**Fig 2:**
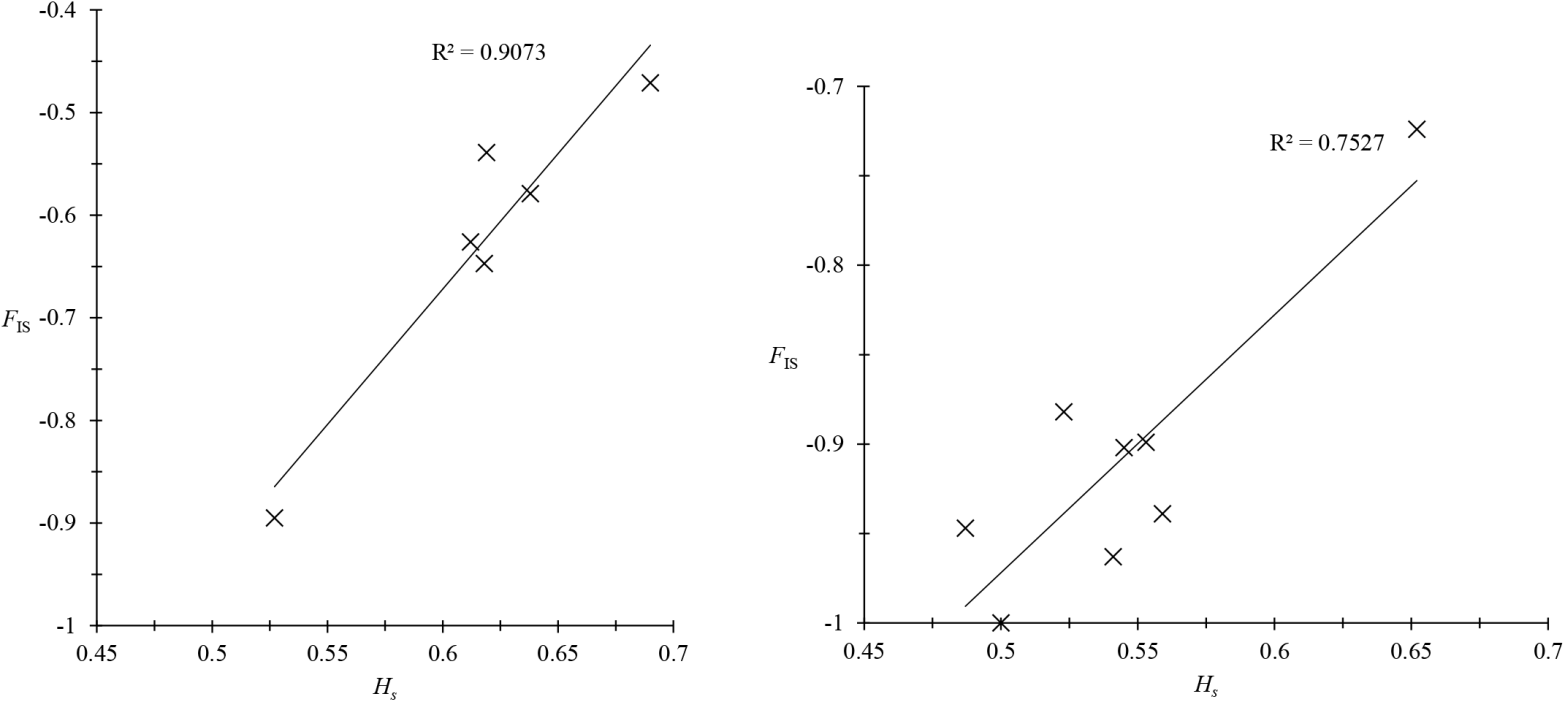
Regression between *F*_IS_, inbreeding index of individuals relative to subpopulations per locus, and Nei's unbiased estimator of genetic diversity *H*_*s*_ [52] in *Trypanosoma brucei gambiense* 1 [19] in West Africa [12] and Central Africa [23]. The proportion of variance explained by the model (*R* ^2^) and the corresponding *p*-values are indicated.

The published results of these analyses concern *T*. *b*. *gambiense* type 1 in West Africa and Central Africa from humans [12, 23] [23] and *T*. *b*. *rhodesiense* in East Africa from humans, cattle, and tsetse flies (Fig 1) [22, 37]. On these isolated stocks of *T*. *b*. *gambiense* type 1, few or no amplification problems occurred at microsatellite loci, and nearly all individuals were heterozygous. In West Africa, out of seven loci (one problematic locus, *Trbpa1/2*, was removed), three individuals were found to be homozygous at locus M6C8 [12]. In Central Africa [23], no homozygotes were found except for 13 at locus M6C8: one in a very small subsample (not used), and 12 in Bipindi as a fixed (no polymorphism) allele that therefore did not influence the *F*_IS_ estimate. Moreover, the variation of the *F*_IS_ from one locus to another was clearly explained by the genetic diversity observed at each locus, and therefore related to the mutation rate specific to each of these loci (Fig 2), as is expected in pure clones [12, 19]. More homozygotes were observed in *T*. *b*. *rhodesiense* minisatellites from humans, especially in the less polymorphic minisatellite (*292*), as expected [17]. No homozygotes were observed in trypanosome minisatellite loci in tsetse flies, while many appeared in cattle (see below).

Across these studies, the demographic parameters of trypanosomes were estimated by assuming that HAT foci are reasonably well isolated from each other. This is true almost everywhere, except possibly in Guinea, where clonal size will appear overestimated by an order of approximately two [12]. Therefore, if we assume isolation between foci, clonal size can be estimated using the following formula [23]:

$$N_{cl}=-\frac{1+F_{IS}}{4uF_{IS}}$$ (Eq. 5)

These clonal sizes were estimated in HAT foci that contained at least five genotyped stocks. The average rates of mutations were assumed to be *u* = 10^−3^ for microsatellite markers [53] (for *u* = 10^−4^ or *u* = 10^−5^ one must multiply these numbers by 10 or 100, respectively) and a rate of *u* = 0.03 for minisatellite markers (see [54], Table 7.5, page 393).

**Fig 3:**
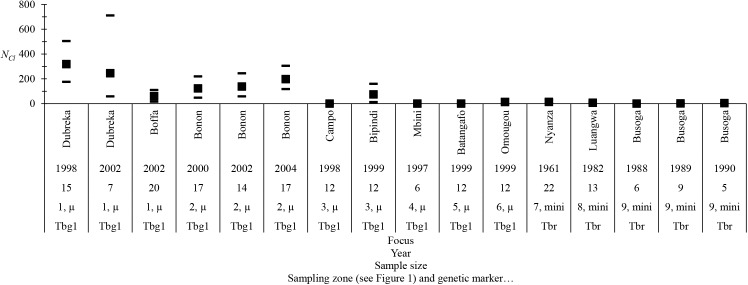
Estimates of clonal population sizes, *N*_*Cl*_ = -(1+*F*_IS_)/(4*uF*_IS_) for an isolated clonal population, of *Trypanosoma brucei gambiense* type 1 (Tbg1) and *T*. *b*. *rhodesiense* (Tbr) infecting humans with a mutation rate *u* = 10^−3^ for microsatellite markers (μ) and *u* = 0.03 for minisatellite markers (mini). The name of the focus, the year of sampling, and the sample sizes are shown on the abscissa. Labels of sampling zones are the same as in Fig 1. For higher mutation rates (e.g., *u* = 0.0001 or 0.00001 for microsatellites), *N*_*Cl*_ values must be multiplied by 10 and 100, respectively.

The results based on these calculations are shown in Fig 3. In this figure, the number of parasites in Dubreka (Guinea) seems lower in 2002 than in 1998; however, the difference is not significant (bilateral Wilcoxon signed rank test, *p*-value = 0.687). Meanwhile, the number of parasites continuously increases in Bonon (Ivory Coast) from 2002 to 2007 (Page test for ordered alternatives, *p*-value < 0.001), despite treatment campaigns conducted for patients before and during this period [55,56]. This result coincides with the beginning of the civil unrest in 2002 in Ivory Coast, with a decrease in the participation rate of the population at risk during medical surveys [56]. In Central Africa, the very low clonal population sizes confirm the low risk of infection in the foci studied as compared to West African foci [57]. Obviously, at least in Cameroon, the prevalence of HAT is very low in most foci, although *T*. *b*. *gambiense* is still found in animals [58]. For *T*. *b*. *rhodesiense* foci, we also noted very low numbers of circulating parasites (*N*_*Cl*_ ≈ 5 ± 4), but this probably stems from an overestimation of the mutation rate. Indeed, *u* = 0.03 does not match the weak genetic diversity found in the available subsamples, especially for locus *292* (*H*_*s*_ = 0.47). If locus *292* is removed, or the mean mutation rate divided by 10, *N*_*Cl*_ of the human *T*. *b*. *rhodesiense* foci fits in the range (14–24) and (12–105), respectively, which seems to more accurately reflect the real situation in terms of human infections [59]. In any case, whether human *T*. *b*. *rhodesiense* or *T*. *b*. *gambiense* 1 is taken into consideration, West African foci seem more dynamical than Central and East African foci, even if the mutation rate of minisatellite loci is substantially decreased (for comparisons across countries and studies within *T*. *b*. *gambiense* 1, the loci are the same). Interestingly, in the cattle subsample of *T*. *b*. *rhodesiense* in the Busoga focus (Uganda), substantial variation of *F*_IS_ was observed across loci (from -0.01 to 0.34 in 1988 and -0.27 to 0.18 in 1990) and across years (from 0.27 in 1988 to -0.02 in 1990), which is the signature of sexual recombination occurring at different rates, depending on the subsample. It is also worth noting that when comparisons were possible (similar dates and sites), there was a strong and significant differentiation between strains sampled from different host species, with a much stronger signal than the differentiation observed between different sampling dates (Table 1). This suggests that trypanosomes circulating in humans and those circulating in animals come from different and genetically distant populations (or even species). Finally, the tsetse fly subsample from Busoga 1969 behaved as a purely clonal population (no homozygotes). This allowed for estimating the clonal population size at *N*_*Cl*_ = 45, with a 95% confidence interval of [4–47]. Nevertheless, the very small sample size (five) makes this purely anecdotal at best.

**Table 1:** Genetic differentiation (*F*_ST_) between *Trypanosoma brucei rhodesiense* subsamples from the Busoga focus (Uganda) according to hosts (human or cattle) and/or year of sampling and significance testing (*p*-value). Data were computed out of three minisatellite loci [37]. When host species is different, only subsamples not separated by more than three years were compared.

Host and year of first subsample	Host and year of second subsample	Time distance in year	*F*_ST_	*p*-value
Cattle 1988	Cattle 1990	2	0.0623	0.0558
Human 1988	Human 1989	1	-0.0017	1
Human 1989	Human 1990	1	0.0702	0.0239
Human 1988	Human 1990	2	0.0600	0.0645
Cattle 1988	Human 1988	0	0.4058	0.0013
Cattle 1990	Human 1990	0	0.2033	0.0001
Cattle 1990	Human 1989	1	0.3223	0.0001
Cattle 1988	Human 1989	1	0.4274	0.0001
Cattle 1988	Human 1990	2	0.2700	0.0052
Cattle 1990	Human 1988	2	0.3057	0.0001

### Non-isolated *T*. *b*. *gambiense*

The study conducted by [60] was a preliminary study carried out using microsatellite DNA amplified directly from biological fluids (i.e., blood, lymph of cervical lymph nodes, and cerebrospinal fluid) to compare the genetic diversity of trypanosomes encountered in these body fluids, as well as to circumvent the in vivo and/or in vitro isolation of trypanosomes.

**Fig 4:**
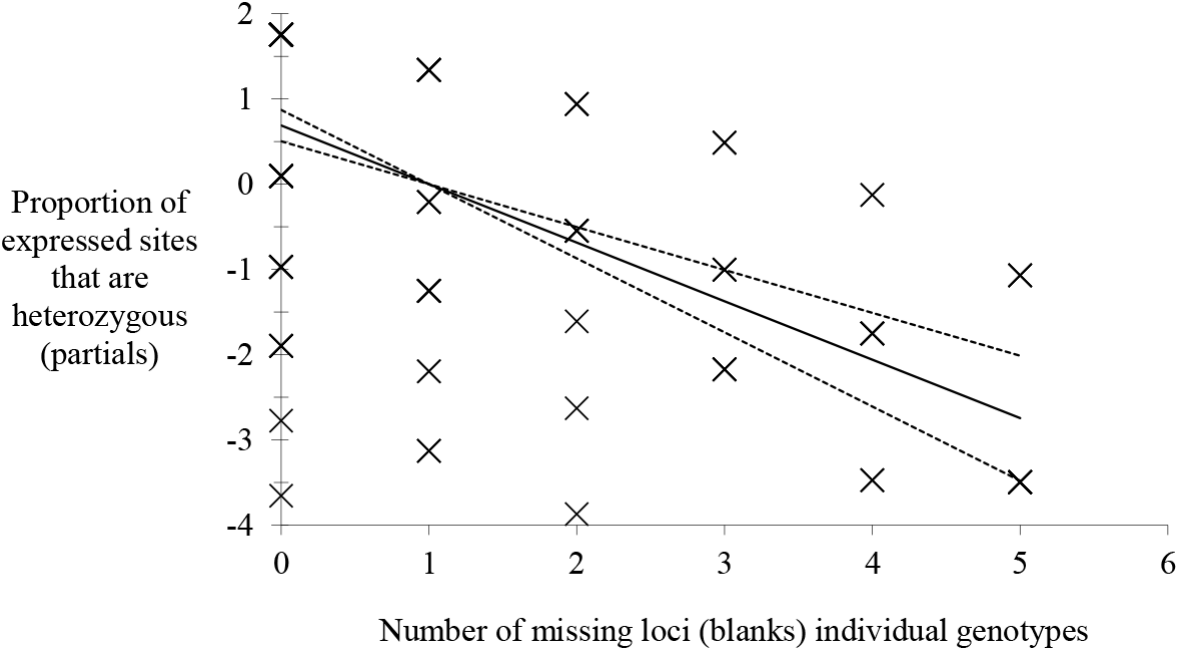
Results of the logistic regression between the proportions of heterozygous genotypes observed in *Trypanosoma brucei gambiense* from Guinea amplified from biological fluids and the number of amplification failures [28]. The relationship has been tested with the chi^2^ test, which proved highly significant (*p*-value < 0.001). 95% confidence intervals are presented with dotted lines.

In this study, an increase in homozygous profiles was observed as compared to previous studies (see above), together with greater heterogeneity of *F*_IS_ between loci. This observation could be diagnostic of recent sexual recombination that may have occurred in the Guinea HAT foci. Nevertheless, a significant number of amplification failures were also observed during this study. Studying the relationship between the proportion of observed heterozygous profiles and these amplification failures, a highly significant negative relationship was revealed (Fig 4), indicating that the higher the number of failures in a complete genotype, the smaller the observed number of heterozygous profiles among expressed loci. This result strongly suggests that a large proportion of "homozygous" profiles observed in this study are in fact heterozygous (one of the two alleles was not amplified as a result of amplification problems) [60]. Moreover, amplifications from lymph nodes in Guinea produced significantly fewer failures than the other fluids (*p-*value < 0.001) [60], because lymph nodes present higher parasitemia in Guinea [61]. Other subsequent experiments demonstrated that most homozygous and many missing genotypes observed by [60] were in fact true heterozygotes [62]. A more recent theoretical approach [19] confirmed that null alleles and/or allelic dropouts can reasonably explain the frequency of homozygous profiles observed in this dataset.

For another dataset on *T*. *b*. *gambiense* [36], loci display a strange behavior with huge variance of *F*_IS_ (from -1 to +0.79). It was shown that under pure clonality, we expect *F*_IS_exp_ = -(1-*H*_S_)/*H*_S_, where *H*_S_ is the unbiased estimator of genetic diversity [19,63]. According to this work, the criterion for significant departure of observed *F*_IS_ *F*_IS_obs_ from the expected *F*_IS_exp_ at one locus in one subsample is when Δ*F*_IS_ = |*F*_IS_obs_-*F*_IS_exp_| ≤ 0.05 × |*F*_IS_obs_-*F*_IS_exp_|. In the other cases, the two values are considered as superimposed. In case of null alleles or rare sex, superimposition decreases. However, it decreases much faster with sex. Here, the proportion of superimposed *F*_IS_ is 50%. This is either compatible with approximately 1% of sex or with 100% clonality and approximately 50% of null alleles (or allelic dropouts) if we refer to the graphic method of [19]. This result is in variance with other observations in similar zones [23]. This should encourage re-genotyping of all homozygous profiles before any useful inference can be made from this dataset.

### Non-isolated *T*. *b*. *rhodesiense*

In Uganda (two sub-samples) and Malawi (one sub-sample), *T*. *b*. *rhodesiense* were sampled at large spatial (69–150 km between the most distant sites) and temporal (two years) scales [38,64]. This was likely to generate spatiotemporal Wahlund effects and, as expected, produced unreliable results. While reanalyzing these data, strong variance in *F* statistics was observed, with a clear positive correlation of *F*_IS_ with the largest distance in the zone considered as a subpopulation by the authors (*R* ^2^ = 0.225, *p*-value = 0.03). This strongly suggests a Wahlund effect. Using the isolates from the smallest area (i.e., Soroti in Uganda, the least affected by Wahlund effects), Sere et al.'s criterion [19] (see above) was compatible with either 99.9% clonality or 100% clonality, and with 20%–50% allelic dropouts or null alleles. Using the same equation as above (Eq 5), with *F*_IS_ = -0.8 in that subsample, we computed *N*_*Cl*_ = 57 (*u* = 10^−3^) with a 95% bootstrap confidence interval of (9–187), which is not far from the number of patients seen (158) [64]. Nevertheless, the high variance across loci and the unicity of the subsample used that extended over one year may prevent further comparisons with other studies. For the other subsamples, some sexual recombination might be occurring, but the data are so heterogeneous that very little can be definitely concluded. Indeed, it is known that the Wahlund effect in clonal organisms can have unpredictable and important effects on population genetic parameters, such as *F*-statistics and linkage disequilibrium [32]. Multiple infections were also observed during this survey. Multiple infections can alter heterozygosity and linkage disequilibrium estimates, and hence have the potential to alter the genetic picture obtained from the population studied. Nonetheless, multiple infections seemed very rare in [38] and probably altered the results very little, if at all.

### Animal trypanosomes

Here, three studies based on direct amplifications from the blood of infected animals gave different results, but all suggested the existence of amplification problems.

**Fig 5:**
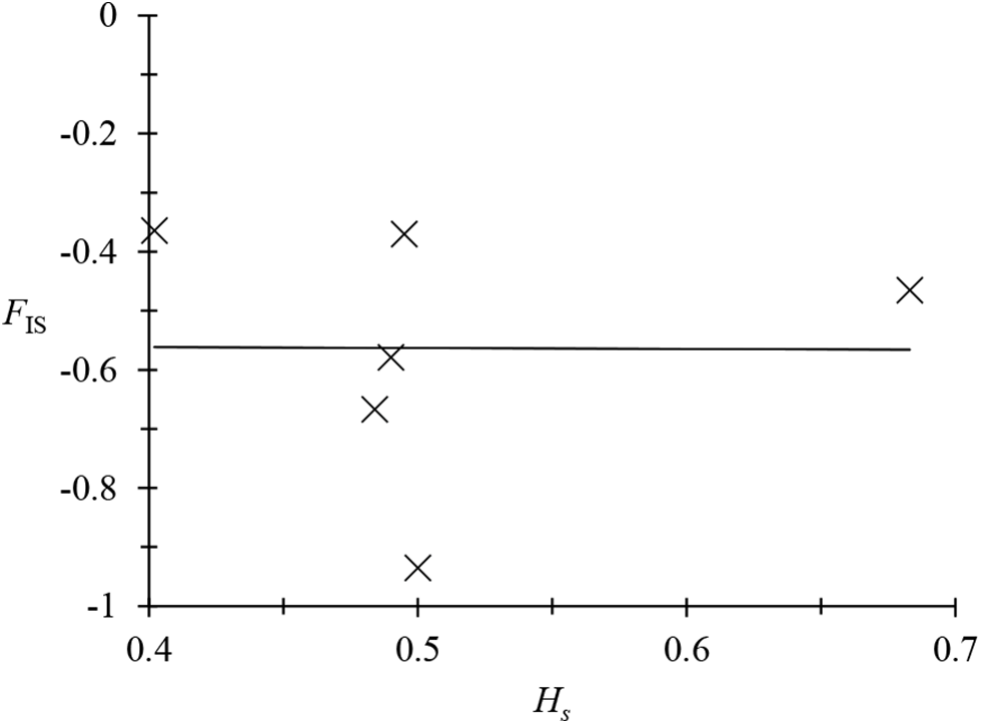
No relationship (*R* ^2^ = 0) between *F*_IS_ per locus and *H*_*s*_ for *T*. *vivax* from The Gambia [5]. The only two loci that seem to behave as expected in a clonal population are connected with a dotted line. These two loci are therefore probably free of amplification problems.

For *T*. *vivax* amplified from cattle, donkeys, and horses in The Gambia [5], reanalysis of the data revealed negative *F*_IS_ that were highly variable across loci [19]. This variability appeared independent from genetic diversity (Fig 5). This is difficult to interpret by the occurrence of rare sex events because no locus showed any *F*_IS_ > 0 (highest *F*_IS_ = -0.4). This can be explained by the existence of amplification failures (allele dropout) that may be more or less frequent depending on the locus considered. Only two loci appeared to have the expected pure clonality profiles and no amplification problems (Fig 5). This would need further analysis to be confirmed, but a more accurate estimate of *F*_IS_ would allow for making demographic inferences. Several multiple infections noted during this survey may add additional problems. Another recent paper [65] on American strains (thus exclusively mechanically transmitted), based on relatively small samples, also found odd results, with *F*_IS_ varying from approximately -1 to +1. This probably also comes from considerable amplification problems.

**Fig 6:**
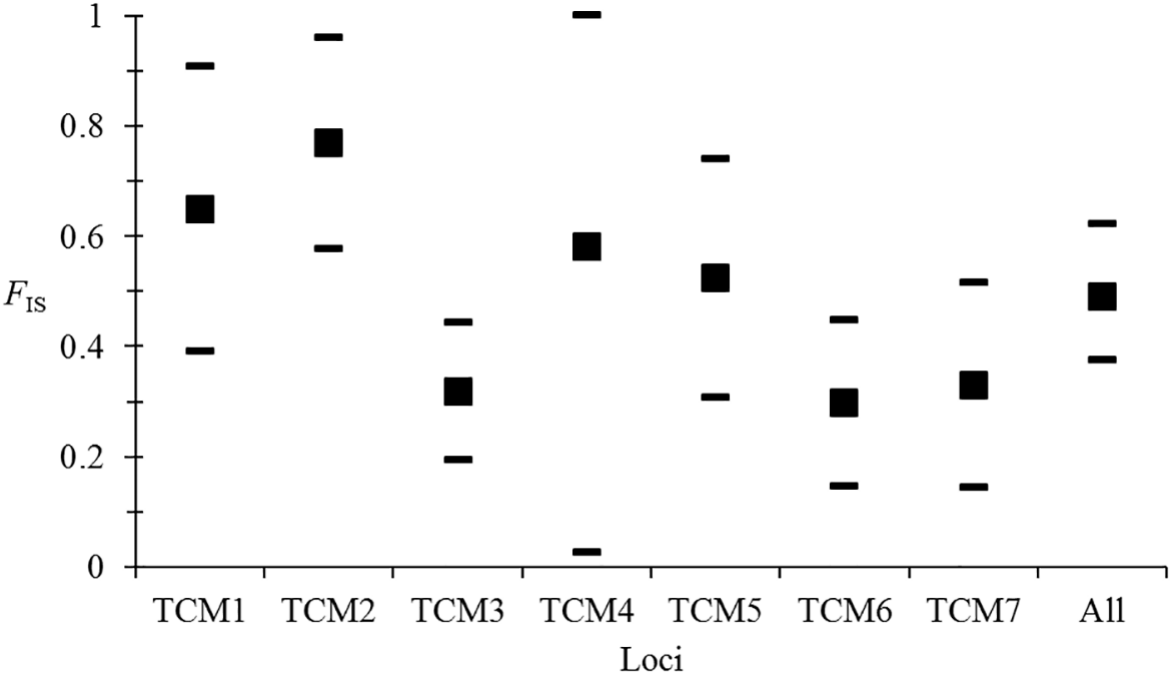
Variation of *F*_IS_ between loci and between subsamples (host species and year) for *Trypanosoma congolense* (“savannah” type) from The Gambia [7]. 95% confidence intervals were obtained by jackknife on subsamples (three host species and two years), except for the average across all loci where the interval was obtained by bootstrapping over loci.

**Fig 7:**
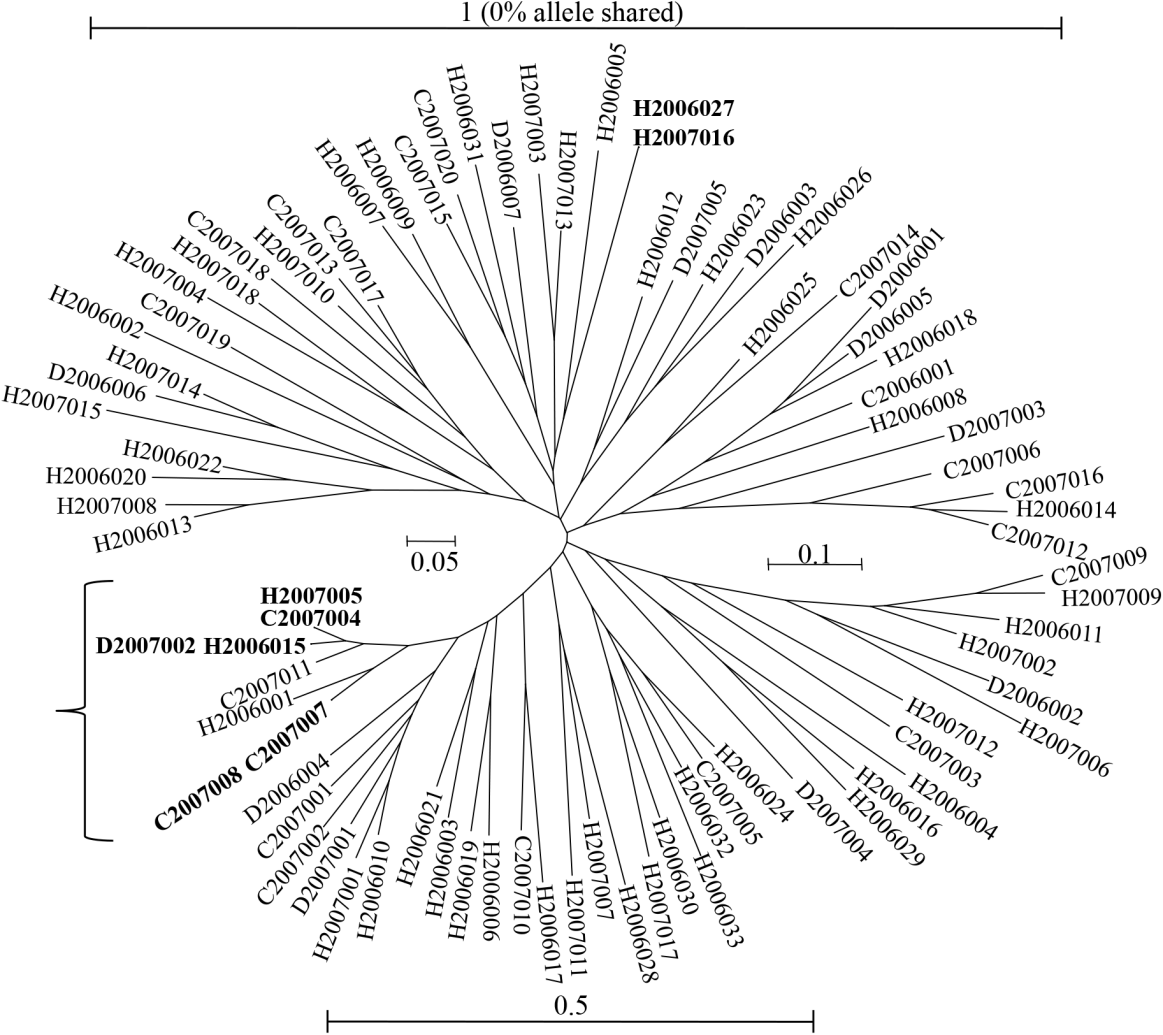
Neighbor-joining dendrogram based on a shared allele distance matrix [67] among pairs of individuals of *Trypanosoma congolense* ("savannah") from The Gambia [7]. The first letter represents the host species (C for cattle, H for horse, and D for donkey) and is followed by the year and finally by the individual numbers. The bracket indicates the most homogeneous group. The genotypes that are identical at all seven loci are shown in bold.

**Fig 8:**
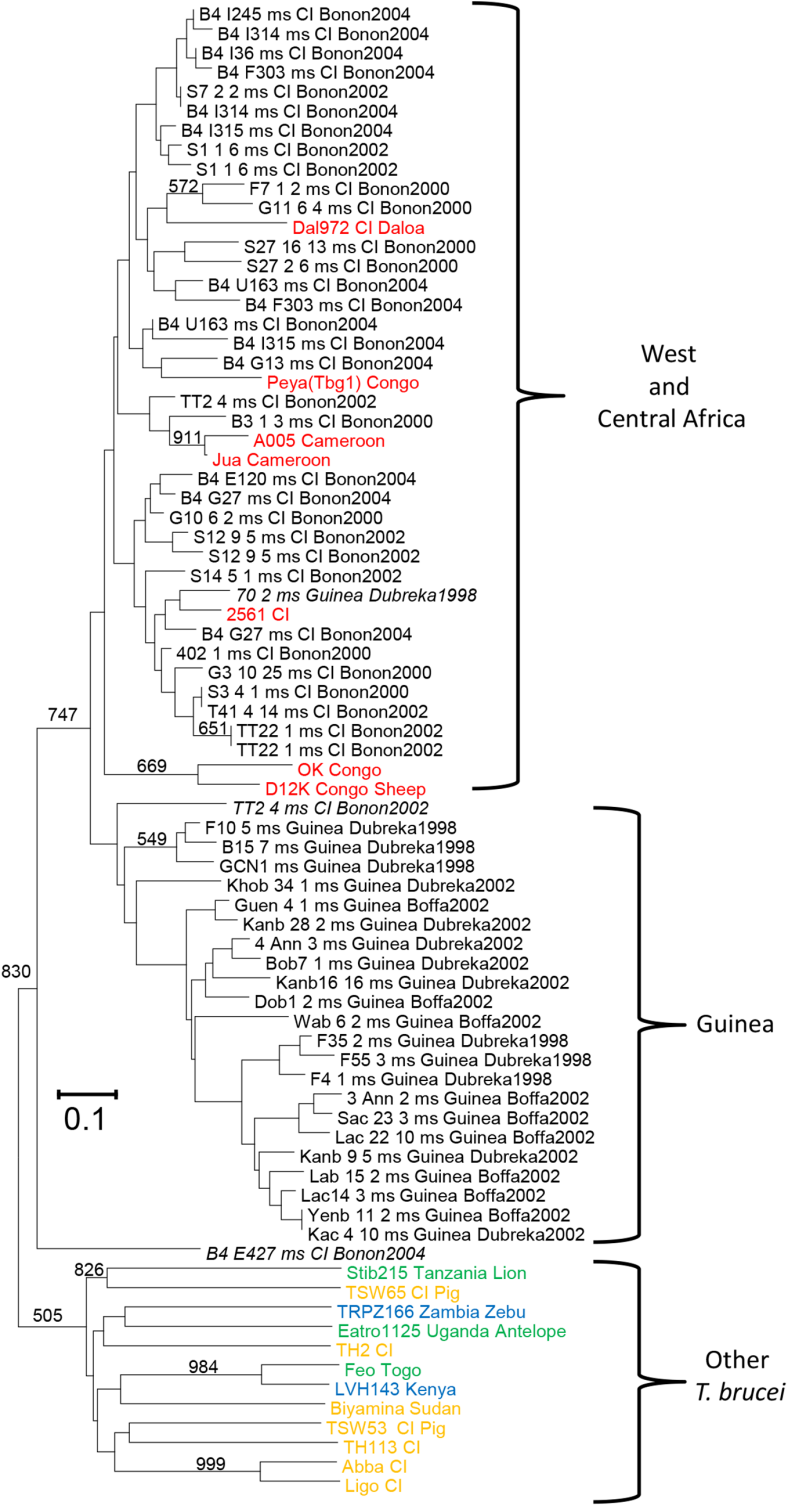
Neighbor-joining dendrogram based on a Cavalli-Sforza and Edwards [80] distance matrix of different samples of *Trypanosoma brucei gambiense* type 1 in Western and Central Africa and computed out of eight microsatellite loci [12]. Red, *T*. *b*. *gambiense* reference strains; gold, *T*. *b*. *gambiense* type 2; green, *T*. *b*. *brucei*; blue, *T*. *b*. *rhodesiense*. Isolates suspected of deriving from immigrants are in italics. Major (>50%) bootstrap values are also indicated. Bootstraps were undertaken with the isolate Stib215 as the root (*T*. *b*. *brucei*).

For *T*. *congolense* (“savannah” type) [7], the samples came from the same host and the same site as in [5]. What is striking is the tremendous homozygosity observed for all loci, but with spectacular variances between loci or between subsamples (host species and year), and a dearth of repeated multilocus genotypes. The authors interpreted these findings as resulting from a combination of the Wahlund effect (mixture of differentiated subpopulations) and frequent sex between related stocks. When reanalyzing the data, this interpretation is difficult to reconcile with the variances observed across loci (Fig 6). Indeed, the Wahlund effect, which is a factor that affects the whole genome homogeneously, and frequent sex (whether inbred or not) cannot generate such variances [8,16]. The second remarkable observation is the great genetic divergences that exist between individual genotypes, as shown in Fig 7. Many genotypes found are indeed almost 100% divergent (according to the seven microsatellite markers used), which is unexpected for individuals of the same species sampled in a relatively narrow space and time. Such distances can be seen between *T*. *b*. *gambiense* and *T*. *b*. *rhodesiense* strains, if we use the shared allelic distance for the data presented in Fig 8. It is worth noting that when analyzing the most homogeneous group in the tree structure shown in Fig 7 (indicated with a bracket), one locus has shown a substantial excess of heterozygotes (*F*_IS_ = -0.273) while all the others nearly exhibited *F*_IS_ = 1. Consequently, this "homogeneous" group can hardly be interpreted as a true entity, because no known reproductive system can generate such a pattern. Finally, the high rate of amplification failures (31%), in addition to all previous observations, and the probable multiple infections, add up to cast doubt on the validity of the genotypes observed. New markers need to be designed and very cautious sampling strategies should be used (at the narrowest spatiotemporal and host species scales). Meanwhile, it seems premature to formulate any inferences on the reproductive system or the population structure of this problematic "species." More recent studies were conducted on strains of the "forest" type from the Fontem focus in Cameroon amplified from mammal blood [66] and tsetse flies [9]. "Savannah" types, although present, apparently could not be genotyped (no data provided). We tried to reanalyze the "forest"-type data. Analysis of *F*_IS_ on contemporaneous subsamples provided results compatible with a substantial amount of sex or clonality with a large proportion of amplification problems (approximately 50%). Frequent missing data, added to obvious cases of multiple strain infections, easily explain the problems encountered, making the data difficult to interpret clearly.

Regarding the samples of *T*. *evansi* isolates from camels in Sudan, an average profile consistent with a panmictic model (*F*_IS_ ≈ 0) was observed [39]. Sexual recombination occurs in tsetse flies’ salivary glands [68,69]. Thus, recombination is highly unlikely for a trypanosome away from any tsetse area where only mechanical transmission occurs [39]. Moreover, *F*_IS_ variance from one locus to another, together with an absence of any repeated genotype, has made it very difficult to interpret such data. The absence of non-amplified genotypes (missing data) dismissed null alleles as a possible explanation. Moreover, random amplification failures (allelic dropout) alone did not seem to explain the data sufficiently. Simulations were then used by the authors to validate the hypothesis that the presence of allele dropout and Wahlund effects (sites contain a mixture of individuals that belong to genetically differentiated populations) may explain the patterns observed. These problems, however, did not prevent the detection of a very significant isolation by distance. It is worth noting that a more recent study that used other loci in Asian *T*. *evansi* samples found data compatible with total clonality in that species (high and invariable heterozygote excesses) [70]. A more recent theoretical study [19] confirmed that allele dropouts can alone explain *T*. *evansi* data from Sudan.

## Discussion

The first, and somewhat frustrating, observation that comes from the overview presented here is the heterogeneity of the datasets in terms of the technique and marker used, with few comparisons possible. Researchers should develop and focus on more robust (in terms of amplification failures) and numerous markers. Highly polymorphic markers should be preferred, because homoplasy will tend to slow the speed at which equilibrium is reached [17]. Ideally, from a logistic point of view, markers that can be amplified directly from body fluids should be preferred. This seems to exclude SNPs for these approaches, but genomic approaches currently being developed will probably provide another perspective regarding reproductive strategies in the long run.

Trypanosome strain isolation techniques are tedious and costly, and present very low success rates [35]. Revisiting many of the available datasets emphasizes that avoiding trypanosome strain isolation steps is possible. However, amplifying parasite DNA directly from the host's body fluids can generate numerous technical problems, probably for the most part due to the small amount of trypanosome DNA available in biological fluids. It will be essential to improve amplification techniques, as proposed in a recent article [62]. Additionally, sampling strategies should target the narrowest possible spatial and temporal scales for each subsample before estimates of ecological parameters can be planned. Multiple infections, when present, will add to the difficulty of interpretation, especially if combined with amplification problems. Indeed, in this case, spurious segregation and recombination can be expected to occur, leading to erroneous interpretations. Multiple infections are encountered in severe infections, together with direct DNA amplification from body fluids. Since only the major circulating strain is amplified through isolation techniques (e.g., RI) [11,12], strain isolation might be a costly cure to this problem. Nevertheless, a less costly technique, yet to be designed, would be welcome, since it is to be expected that many more problematic datasets containing such problems will be gathered in the near future, in particular with genomic approaches.

The clonality of *T*. *b*. *gambiense* 1 is now a well-established fact [12,19], at least in a mid- to short-term perspective. For *T*. *vivax* and *T*. *evansi*, this also seems to be the case, despite imperfect data; but further studies with better genetic markers will be required for confirmation. For other taxa, taxonomic heterogeneity, together with DNA amplification problems and sampling difficulties, or even absence of reliable data, include enough confounding factors that prevent definitive conclusions from being drawn for any of them. We know that sexual recombination is possible within and between different laboratory strains of *T*. *b*. *brucei*, *T*. *b*. *gambiense* 2, and *T*. *b*. *rhodesiense* (and never *T*. *b*. *gambiense* 1) in tsetse fly salivary glands [2,71]. We also know that these three taxa are composed of different and divergent entities ([72,73]; see also below). Some distant members of these lineages are also suspected of having undergone hybridization events in the past [74]. The importance of recombination in wild conditions and in these different lineages thus remains an important route for further investigation. *T*. *congolense*, *T*. *vivax*, and *T*. *evansi* do not colonize tsetse flies’ salivary glands [5–7]. Although absence of sexual recombination could be predicted for these taxa, this remains to be determined with appropriate sampling and tools. Escaping the tsetse belt means that new environments and new hosts can be colonized. This provides an advantage that might have occurred several times independently in different lineages of *T*. *b*. *brucei* [75]. In the absence of possible sexual recombination with the tsetse fly salivary gland, this naturally would have led to the propagation of different asexual lineages of *T*. *evansi* and *T*. *equiperdum*. If these different lineages can coexist in the same environments, an interesting track of investigation remains to be undertaken.

When estimates are possible, clonal population sizes appear relatively consistent or slightly higher than what medical surveys suggested. Nevertheless, for microsatellite markers, such estimates were undertaken with the assumption of a very important averaged mutation rate for microsatellite loci (*u* = 0.001). There is evidence, however, that mitosis generates less frequent mutations and that the mutation rate could be as low as 0.00001 for microsatellite loci in clonally propagating populations [19]. This would provide a pessimistic picture of the current epidemiological state of sleeping sickness, especially in West African foci where tens of thousands clones would be expected to circulate in hidden human and/or animal reservoirs. In a context in which the elimination of the disease is considered as a reasonable target by the WHO, which has projected fewer than 2,000 reported cases per year and more than 90% foci with less than one case per 10,000 inhabitants [76], clarifying this issue is an important goal. More analyses on more samples are therefore needed.

Another emerging feature of these studies is the tremendous genetic heterogeneity within a taxon that can be observed in many studies, especially (but not only) in *T*. *congolense*. These heterogeneities suggest the existence of subdivisions into very small clusters (subsets) or even the existence of different species that remain to be characterized. As already mentioned in a previous article [77], the *T*. *brucei* complex should inspire us to rethink the taxonomy of these parasites of medical and veterinary importance. According to Fig 8, two clusters can be distinguished (though with a low level of bootstrap support) in *T*. *b*. *gambiense*: one from Guinea, transmitted by *Glossina palpalis gambiensis* in mangrove areas with a high lymphatic tropism, and one from Ivory Coast and Central Africa, transmitted by *G*. *p*. *palpalis* in forest areas with high blood tropism. Some more or less recent immigration signatures can be suspected based on Fig 8. A Dubreka strain (isolate 70 2ms Guinea Dubreka 1998) has been observed within the West and Central Africa "clade," and a Bonon strain has been observed within the Guinean "clade" (TT2 4ms CI Bonon 2002). Furthermore, a possible hybrid between a *T*. *b*. *gambiense* strain and a non-*gambiense T*. *brucei* (B4E427 msCI Bonon 2004) has also been observed. The possible existence of hybrids will need to be investigated further, because they could provide evidence of the (very) rare occurrence of sexual recombination in *T*. *b*. *gambiense*. Using the divergence measured between the two alleles of each hemigenome (Meselson effect [78,79]), genomic studies should confirm long-term clonality and also provide a tool to estimate when *T*. *b*. *gambiense* 1 became totally clonal.

We will not dwell on the cases of *T*. *b*. *rhodesiense* (see also [81]), *T*. *b*. *gambiense* group 2, or *T*. *b*. *brucei* whose heterogeneity (Fig 8) barely hides the probable existence of species complexes that remain to be deciphered and are likely related to ecological differences (host and/or landscape). This is not a purely academic consideration, given the economic and public health significance of these human and animal parasites from sub-Saharan Africa, and will require further scientific investigations.

Finally, some of the issues discussed above will find new answers with the emergent use of genomic approaches and analyses of SNPs on a large scale. Nevertheless, to date, these approaches still need tedious and costly isolation of parasite isolates. SNPs display maximum homoplasy and will thus reach expected equilibrium values with a much higher number of generations (more than 20,000) [17]. Because of the huge number of markers involved, wide-scale genomic studies will meet the difficulty of handling markers with a heterogeneous determinism, from purely neutral to highly selected, with little opportunity for screening the most neutral markers. Because SNP mutation rates are also expected to be extremely low in clones, the signature of selective events can never be wiped out before a prohibitive number of generations. For these reasons, microsatellite markers (especially dinucleotide markers, because of their non-coding nature) will remain the markers of choice for studying the (short-term) population genetics of trypanosomes, owing to high polymorphism and high mutation rates, and because they also offer the best opportunity for direct amplification from body fluids.

**Box 1. Key Learning Points**

1. Direct amplification of parasite DNA from body fluids (blood, lymph, or cerebrospinal fluid) allows for study of the population genetics of African trypanosomes, by bypassing the costly and tedious strain isolation steps. Nevertheless, two problems remain to be solved: the existence of amplification problems, mainly due to small concentrations of trypanosome DNA, and multiple infections. This may blur the true genotypes of individuals and even generate spurious recombinations. In some cases, this jeopardizes the population genetics inferences that can be extracted from available current data.
2. Except for the monophyletic *Trypanosoma brucei gambiense* type 1, taxa belonging to the subgenus *Trypanozoon* (*T*. *b*. *gambiense* type 2, *T*. *b*. *brucei*, *T*. *b*. *rhodesiense*, *T*. *evansi*, and *T*. *equiperdum*) are composed of divergent lineages. Some of these lineages probably display different ecological needs that remain to be described. This might unveil particular features that might prove useful in some instances (e.g., control).
3. Sexual recombination can only occur in tsetse salivary glands, and should therefore be absent from trypanosomes that avoid this step, such as *T*. *evansi*, *T*. *congolense*, *T*. *vivax*, and *T*. *equiperdum*. This awaits confirmation based on relevant sampling with flawless markers. Sex seems absent from *T*. *gambiense* type 1, even if some old and rare hybridization events might have occurred in the past, which remains to be confirmed. For *T*. *b*. *gambiense* type 2, *T*. *b*. *brucei*, and *T*. *b*. *rhodesiense*, appropriate sampling with flawless markers and homogeneous subsamples in time and space will teach us more about the biosytematics of these complex lineages and about the frequency with which sex occurs in the different lineages that compose these three taxa.
4. In lineages known to be fully clonal, estimating clonal size of subpopulations and immigration from neighboring sites (foci) is possible. For lineages displaying some recombination, unless if frequent enough (e.g., approximately 50%), inferences might prove problematic.

**Box 2. Top Five Papers**

1. Balloux, F., Lehmann, L., De Meeûs, T. 2003. The population genetics of clonal and partially clonal diploids. Genetics 164, 1635–1644.
2. De Meeûs, T., Lehmann, L., Balloux, F. 2006.Molecular epidemiology of clonal diploids: A quick overview and a short DIY (do it yourself) notice. Infect. Genet.Evol. 6, 163–170.
3. Gibson, W.C. 2007. Resolution of the species problem in African trypanosomes. Int. J. Parasitol. 37, 829–838.
4. Koffi, M., De Meeûs, T., Bucheton, B., Solano, P., Camara, M., Kaba, D., Cuny, G., Ayala, F.J., Jamonneau, V. 2009. Population genetics of *Trypanosoma brucei gambiense*, the agent of sleeping sickness in Western Africa. Proc. Natl. Acad. Sci. U. S. A. 106, 209–214.
5. Balmer, O., Beadell, J.S., Gibson, W., Caccone, A. 2011. Phylogeography and Taxonomy of *Trypanosoma brucei*. PLoSNegl. Trop. Dis. 5, e961.
